# Laboratory strains of *Bacillus anthracis* lose their ability to rapidly grow and sporulate compared to wildlife outbreak strains

**DOI:** 10.1371/journal.pone.0228270

**Published:** 2020-01-24

**Authors:** Michael H. Norris, Diansy Zincke, Owen P. Leiser, Helen Kreuzer, Ted L. Hadfied, Jason K. Blackburn

**Affiliations:** 1 Spatial Epidemiology & Ecology Research Laboratory, Department of Geography, University of Florida, Gainesville, Florida, United States of America; 2 Emerging Pathogens Institute, University of Florida, Gainesville, Florida, United States of America; 3 Chemical and Biological Signature Science, Pacific Northwest National Laboratory, Richland, Washington, United States of America; Spectrum Health, UNITED STATES

## Abstract

*Bacillus anthracis* is the causative agent of anthrax in animals and humans. The organism lies in a dormant state in the soil until introduced into an animal via, ingestion, cutaneous inoculation or inhalation. Once in the host, spores germinate into rapidly growing vegetative cells elaborating toxins. When animals die of anthrax, vegetative bacteria sporulate upon nutrient limitation in the carcass or soil while in the presence of air. After release into the soil environment, spores form a localized infectious zone (LIZ) at and around the carcass. Laboratory strains of *B*. *anthracis* produce fewer proteins associated with growth and sporulation compared to wild strains isolated from recent zoonotic disease events. We verified wild strains grow more rapidly than lab strains demonstrating a greater responsiveness to nutrient availability. Sporulation was significantly more rapid in these wild strains compared to lab strains, indicating wild strains are able to sporulate faster due to nutrient limitation while laboratory strains have a decrease in the speed at which they utilize nutrients and an increase in time to sporulation. These findings have implications for disease control at the LIZ as well as on the infectious cycle of this dangerous zoonotic pathogen.

## Introduction

Anthrax, caused by the spore forming bacterium *Bacillus anthracis*, is an important and under reported disease. Naturally occurring, anthrax causes epizootics in wildlife, livestock (including mixed wildlife/livestock outbreaks) and is associated with spillover to adjacent human populations [1]. Spores are found in specific soil conditions nearly worldwide and are infectious to many species of animals, primarily grazing and browsing ruminants, often resulting in high mortality [2]. Spores are reported to have a half-life of approximately 100 years [3] and environmental decontamination is difficult [4]. The current hypothesis for anthrax transmission states that carcasses from anthrax deaths form localized infectious zones (LIZs) at the carcass site (and immediate vicinity) where future infections occur during grazing or bone chewing [1,5]; flies may expand these zones in the short-term [6–8]. Veterinary vaccination programs are the most effective control mechanism but decontamination of carcasses when vaccination is untenable, such as in wildlife outbreaks [9,10] is also a good control measure.

Recently our group found that frequently passaged laboratory strains of *B*. *anthracis*, including sub-lineages Ames, Sterne, Vollum (A4), Western North America (WNA; A1.a) have very different protein profiles compared to low passage “wild” strains of similar sub-lineages isolated from recent wildlife, livestock, and mixed (livestock/wildlife) outbreaks in Montana, Colorado, and Texas [11]. Principal component analysis (PCA) separated wild strains of *B*. *anthracis* from lab strains based on global protein abundance comparisons. Many proteins involved in sporulation were more abundant in wild strains when compared to long-term laboratory strains, suggesting laboratory strains adapt quickly to high-nutrient growth media and become less efficient at sporulation. Conversely, the data implied wild strains are more attuned to rapid sporulation in response to nutrient depletion.

An important part of the *B*. anthracis lifecycle that is often overlooked is environmental persistence. Besides virulence and host-infection, it is the other half of the pathogen lifecycle. Sporulation is a vital step in *B*. *anthracis* survival because the vegetative cell is easily outcompeted by other organisms and is very fragile under normal environmental conditions [12]. Understanding how wild strains differ from laboratory strains in their ability to survive harsh conditions and perpetuate outbreaks is a goal of our laboratory. Here we measured growth and sporulation rates of standard laboratory strains and natural isolates of multiple molecular groups of *B*. *anthracis*. *B*. *anthracis* physiology and ecology studies utilize type strains, such as Ames and Sterne, that have been grown in the lab for decades, potentially generating misleading data if extrapolated to observations of persistence in nature.

## Materials and methods

### Bacterial strains and growth conditions

We used the same strains that were previously used for intensive proteomic profiling [11]. These strains are archived in the Martin E. Hugh-Jones *Bacillus anthracis* Collection at the University of Florida and handled in a CDC/USDA registered and inspected BSL3 facility according to the practices and procedures recommended by the Biosafety in Microbiological and Biomedical Laboratories 5^th^ edition [13]. Geographically and temporally distinct wild strains of *B*. *anthracis* were isolated from wildlife during outbreak investigations in the western United States (white-tailed deer, *Odocoileus virginianus*, 2004, 2009, Texas; bison, *Bison bison bison* and elk, *Cervus canadensis*, 2008, Montana). In 2012, an additional strain was isolated from a domestic cow in northwestern Colorado [14]. Strains are summarized in Table 1. Wild strains were genotyped using the 25 marker multi-locus variable number tandem repeat (MLVA) described by Lista et al. [15] and reported elsewhere [16]. All wild isolates were cultured on 5% sheep blood tryptic soy agar (SBA) no more than three times while “lab” strains were cultured an unknown number of times but realistically greater than 100 passages. Frozen stocks created from SBA colonies are stored in Tryptic Soy Broth (TSB) with 10% glycerol at -80°C.

**Table 1:** Details of *B*. *anthracis* strains used in this study.

UF* Strain ID	Lab vs. Wild	Related laboratory strain	Strain details	Outbreak details	pXO1/ pXO2 ‡	Genotype designation^†^	References
Ba553	Lab	Sterne-Lab	NA	NA	+/-	48	[17]
Ba738	Lab	Ames-Lab	NA	NA	+/+	53	[18]
Ba980	Lab	Vollum-Lab	NA	NA	+/+	54	[19]
Ba147	Lab	WNA-Lab	NA	NA	+/+	39	[20]
Ba1114	Wild	Sterne^a^	2009 Texas deer	2 white-tailed deer died in a coastal pothole in south Texas	+/-	48	[16]
Ba1105	Wild	Ames^b^	2009 W. Texas deer	Large ranch-wide white-tailed deer outbreak in West Texas	+/+	53	[16]
Ba1106	Wild	Ames^b^	2009 W. Texas deer	Large ranch-wide white-tailed deer outbreak in West Texas	+/+	53	[16]
Ba1096	Wild	Vollum (A4)	2004 W. Texas deer	Sporadic white-tailed deer case on a well-studied ranch	+/+	54	[16,21]
Ba1103	Wild	Vollum (A4)	2009 W. Texas deer	Large mixed livestock/wildlife outbreak; strain used here from a white-tailed deer	+/+	54	[8,16]
Ba1137	Wild	WNA (A1.a)	2012 Colorado cow	Single isolate from a domestic cow involved in an outbreak in northeastern Colorado; first outbreak confirmed in the area since the 1970s	+/+	39	[22]
Ba1043	Wild	WNA (A1.a)	2008 Montana elk	large mixed bison, elk, white-tailed deer outbreak in Western Montana	+/+	2	[10,16]

^a^MLVA-based genotype relates to Sterne based on lack of pX02 plasmid

^b^Ames-like lineage but not true Ames

*UF = University of Florida

^‡^ = pXO1/pXO2 status verified as previously described [8].

^†^ = genotype according to MLVA-25 typing [15] and [16].

### *Bacillus anthracis* sporulation characterization and sporulation rate determination

For this study, strains were streaked onto BHI agar and incubated at 30°C for 24–48 h. Several colonies were picked and resuspended in PBS with 0.05% Tween-20 (PBST). The cell suspension was diluted in sterile saline and the OD_600_ of the original suspension was determined using an Ultrospec 10 Cell Density Meter (Amersham). The bacterial suspension was adjusted to an OD_600_ of 1 with PBST. The OD_600_ of 1 PBST cellular suspension was used to inoculate 2 ml of BHI broth in 0.22 μm ventilated cap 15 ml tubes (CELLTREAT Scientific Products) at a 1:100 dilution. Each strain was inoculated in triplicate then incubated at 30°C in a shaking incubator at 220 rpm. We chose 30°C as the temperature for growth in our assays because it is a warm ambient temperature and was previously found to be optimal for *B*. *anthracis* survival in soil environments [23,24]. BHI broth is essentially digested animal tissues and serves as a closer simulacrum to an animal carcass than the various nutrient poor sporulation media utilized in the literature.

At 24 and 48 h, two 50 μl aliquots were removed. One aliquot was serially diluted in PBST and plated on BHI agar to determine total colony-forming units (CFU). The second aliquot was treated with 90% ethanol for 1 h then diluted in PBST and plated on BHI agar to determine the spore count. Ethanol treatment has been shown to be equivalent to 65°C heat-shock for removing vegetative cells from mixed vegetative/spore suspensions [25]. In our hands the ethanol soak method has proven superior to 65°C heat-shock for recovery of all viable spores. The dilution plates were incubated at 30°C and counted 24–48 h later. This experiment was carried out 3 times with three biological replicates each. Data from one experiment is presented for clarity. The sporulation rate (spores/min) of the various strains were determined from the slope of the lines between the 24 h and 48 h timepoints of each strain divided by 60 minutes. The rates for each biological replicate were averaged and standard deviations were calculated. Lab strains and wild strains were grouped, and the non-parametric Mann-Whiney U test was used to determine significant differences between groups using the GraphPad Prism software.

### Plate-based growth assay and doubling time calculation

Bacterial cultures were grown overnight in BHI Broth then diluted to an OD_600_ of 1 in BHI. The dilution was used to inoculate fresh BHI at a 1:100 dilution and the OD_600_ read every 2 h in a Tecan Sunrise plate reader incubated at 37°C. Doubling time was determined from OD_600_ measurements at the beginning and end of exponential growth. Sigmoidal interpolation was carried out using Graph Pad Prism software to identify the beginning and end of exponential growth. Growth rates (r) were calculated by the formula r = (ln [OD2/OD1]) / (T2-T1) and the doubling time corresponds to ln(2)/r.

## Results

**Fig 1:**
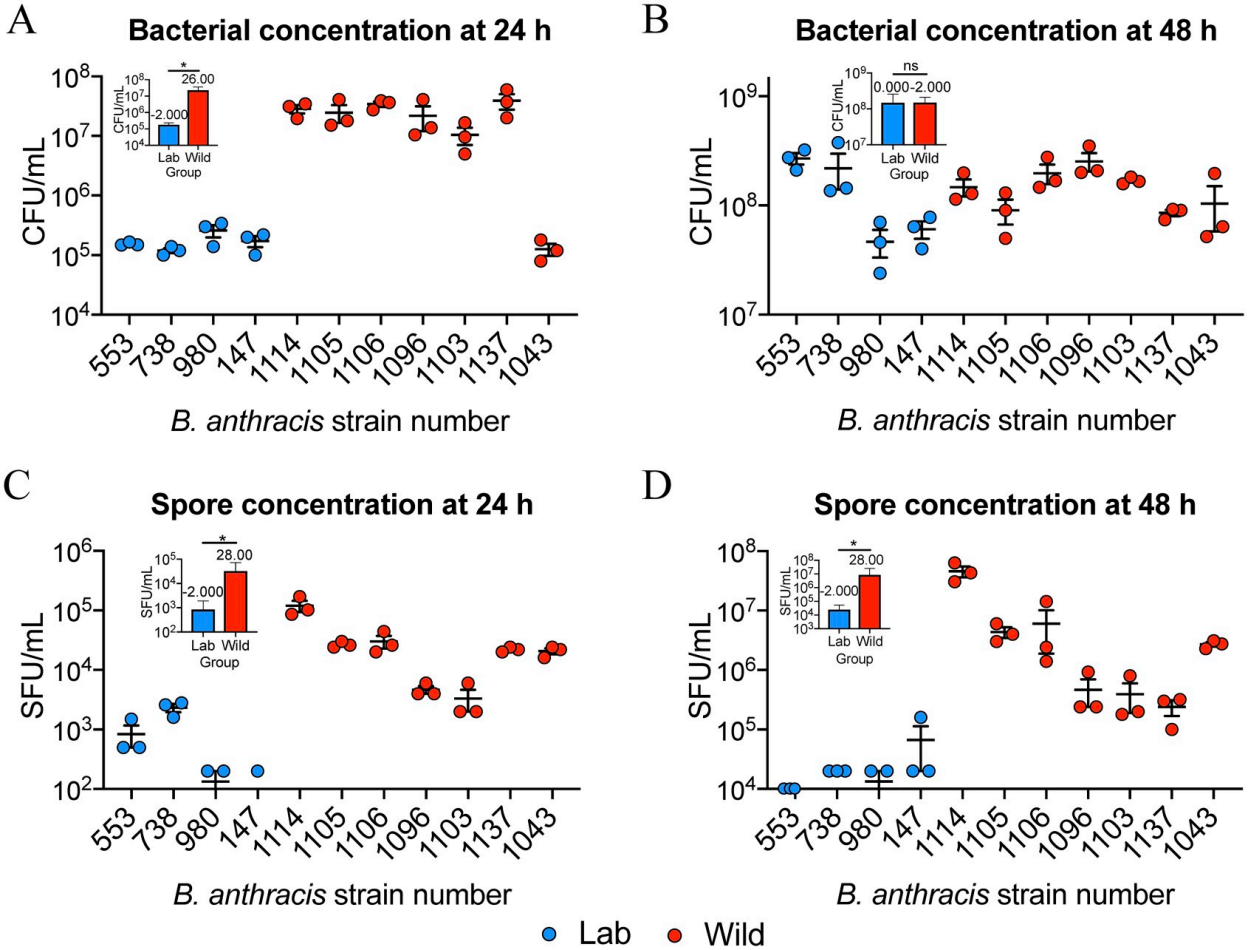
Bacterial and spore concentrations of lab and wild grown *B*. *anthracis*. A) CFU/ml of lab strains (blue) and wild strains (red) at 24 and 48 h (C). B) Spore forming units/ml (SFU/ml) of the same cultures at 24 and 48 h (D). Data points are the CFU/ml or SFU/ml of experimental triplicates. The error bars are the standard deviation. Insets are Mann-Whitney U tests of lab and wild strains as groups, U (lab) and U’(wild) are listed above the blue and red bars, respectively, and * *p < 0*.*05; ns* = not significant.

**Fig 2:**
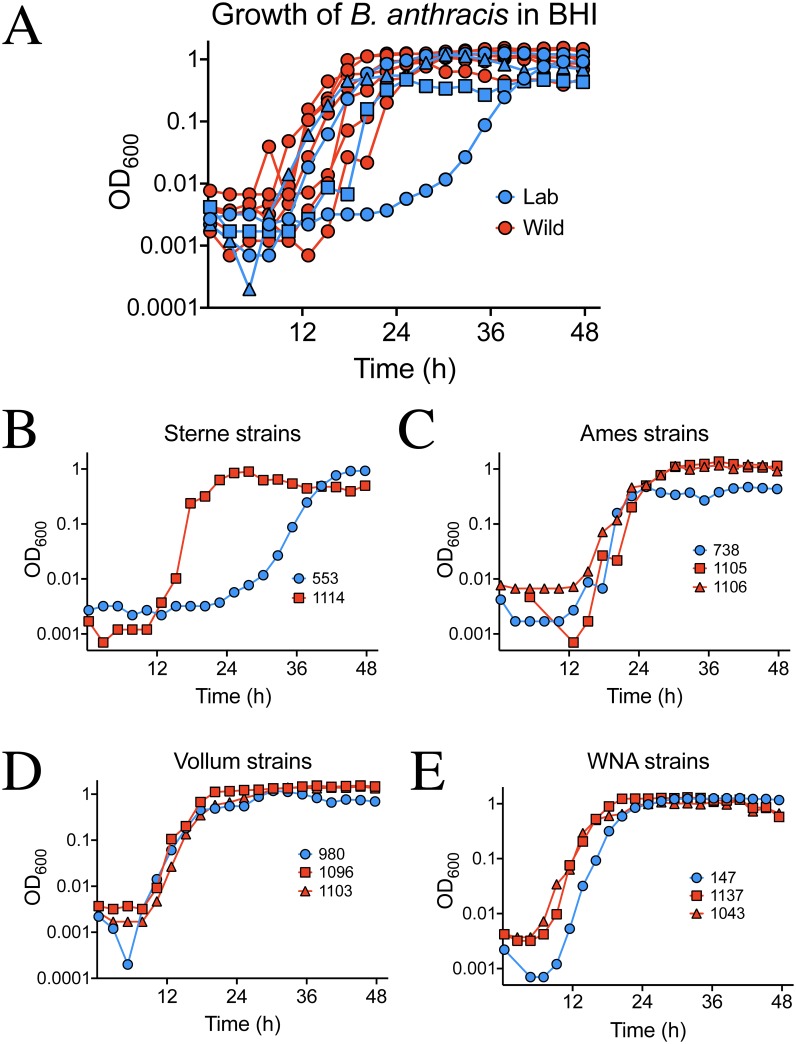
Growth analysis of wild and lab strains of *B*. *anthracis*. Growth rate analysis of *B*. *anthracis* was carried out in a Tecan Sunrise 96-well plate shaking incubator in BHI broth. A), Growth curves of all strain data. Red curves indicate wild strains of *B*. *anthracis* and blue lines indicate common laboratory strains. B), Our wild Sterne-like *B*. *anthracis* strain UF01114 (red) that is pXO2 ^−^ has a much shorter lag-phase and enters log phase well ahead of our Sterne laboratory strain (blue). In C), the wild Ames-like strains (red circles and red triangles) achieve a higher final cell density than our Ames laboratory strain (blue). In D), a wild Vollum-like strain, UF01096 (red circles), has a higher rate of logarithmic growth compared to our Vollum lab strain (blue circles). Strain, UF01103 (red triangles), grows the same as the Vollum-lab. In E), our Western North American (WNA) wild strains (red circles and triangles) enter logarithmic growth earlier and at a steeper rate than WNA lab strain UF00147 (blue). The mean of two OD_600_ measurements every 2 h are shown.

Wild *B*. *anthracis* reached higher cell densities than laboratory-adapted strains after 24 hour growth. At this time point, all laboratory adapted strains had grown to ~10^5^ CFU/ml while, with the exception of one, wild strain cell densities were ~100 times greater at ~10^7^ CFU/ml (Fig 1A; S1 Table). Strain 1043, isolated in 2008 from a dead elk in Montana, displayed cell densities more similar to those of the laboratory adapted strains. Even so, as a group, wild strain cell densities were significantly higher than those of laboratory adapted strains at 24 h as determined by the Mann-Whitney U test (Fig 1A inset). By 48 h, all strains had reached nearly 10^8^ CFU/ml, and there was no significant difference between laboratory adapted and wild groups (Fig 1B and 1B inset). This data suggested that wild strains of *B*. *anthracis* grew faster than the laboratory-adapted strains. Slower growth of the laboratory-adapted strains was verified in 96-well plate-based growth assays (Fig 2; S2 Table).

Spores were enumerated at 24 and 48 h because complete sporulation occurs by 72 h in laboratory-adapted cultures [23,26]. Spore concentrations at 24 h were ~5–100 times higher in wild strains versus laboratory-adapted strains (Fig 1B). The highest concentrations of spores were found in a wild Sterne-like strain (UF01114) isolated in 2009 from the carcass of a Texas deer and the lowest were in Sterne (laboratory adapted strain UF00147). The next highest were from the wild Ames-like (UF01105 and UF01106) and WNA strains (UF01137 and UF01043). Strain UF01043 grew slowly but had higher spore levels as a percentage of total CFU relative to the other wild strains at 17.4% (Table 2). Wild strains UF01096 and UF01103, both Vollum-like strains isolated from Texas deer, had the lowest 24 h spore concentration. In the previous proteomics study these two strains were clustered near one another in a PCA [11] and behaved similarly here. Comparison of laboratory adapted and wild strains as groups showed wild strains formed significantly more spores at 24 h than lab strains (Fig 1C inset). Spore concentrations at 48 h remained significantly higher in wild cultures compared to laboratory-adapted cultures (Fig 1D insert). In terms of both sporulation rate and spore percentage of total CFU, Sterne-like *B*. *anthracis* strain UF01114 was the most efficient sporulator between the timepoints (Table 2). All laboratory adapted strains’ spore concentrations were lower than those of the wild strains (Fig 1D). Plate-based growth curves were utilized to verify observed growth characteristics (Fig 2A–2E). Wild strains had reduced lag phases (Fig 2B and 2E), higher growth rates (Fig 2D and 2E), higher final optical densities (Fig 2B and 2C) or entered stationary phase (Fig 2B, 2C and 2E) before wild strains when compared within lineages.

**Table 2:** Sporulation rates and average spores after growth at 30°C in BHI.

Sporulation rates of *B*. *anthracis* lab and wild strains		Average % spores (SFU/CFU x 100)^c^	
*B*. *anthracis* strain^a^	Sporulation rate (spores/min)^b^	24 h	48 h**
**553 (Sterne)** **[UF00553]**	−0.58 ± 0.23	0.52	0
**738 (Ames)** **[UF00738]**	12.26 ± 0.26	1.92	0.01
**980 (Vollum)** **[UF00980]**	9.17 ± 4.63	0.07	0.04
**147 (WNA)** **[UF00147]**	46.25 ± 32.4	0.07	0.11
**1114** **[UF01114]**	31676.38 ± 6634	0.43	34.29
**1105** **[UF01105]**	2990.73 ± 612.45	0.13	5.25
**1106** **[UF01106]**	4145.83 ± 2854.28	0.09	3.89
**1096** **[UF01096]**	320.83 ± 157.4	0.03	0.21
**1103** **[UF01103]**	270.83 ± 141.27	0.05	0.24
**1137** **[UF01137]**	151.38 ± 48.78	0.07	0.29
**1043** **[UF01043]**	1865.28 ± 164.8	17.41	3.80

^a^ Strains in blue are lab strains and red are wild strains. Parenthesis indicate common names of lab strains. Strains are identified by their bracketed accession number in the Martin E. Hugh-Jones collection.

^b^Data are spores/min between 24 and 48 h including plus or minus the SD of the 3 replicate experiments.

^c^For ease of comparison averages are presented here.

**Wild strains had significantly higher percentages of spores at 48. *p*<0.01 by the Mann-Whitney U test.

Sporulation rates were calculated as the number of spores formed between 24 h and 48 h divided by 1440 minutes. The average sporulation rates for all wild strains were greater than 151 spores/min with the maximum of 32,676 spores/min measured in the Sterne-like strain from the Texas deer carcass (UF01114) (Table 2, red strains). *Ba* strain UF01114 also had the most spores as a percentage of total CFU at 34.29% by 48 h. Average sporulation rates of laboratory adapted strains were below 50 spores/min and for Sterne (UF00147), spores were not detected in two of three replicates at 24 h (Table 2, blue strains).

Culture doubling times calculated from plate-based growth experiments showed all wild strains but UF01103 had faster doubling times during exponential growth than lab strains (Table 3). If the length of lag time is ignored, and focus is placed solely on the doubling time, we can see that after the lag phase, wild strains grow faster in log phase (Table 3). These data confirmed strain performance in the tube-based CFU density experiments.

**Table 3:** Doubling time of *B*. *anthracis* lab and wild strains at 30°C.

*B*. *anthracis* strain^a^	Doubling time (min)^b^
**553 (Sterne)** **[UF00553]**	203.67 ± 126.84
**738 (Ames)** **[UF00738]**	249.87 ± 18.75
**980 (Vollum)** **[UF00980]**	300.93 ± 29.74
**147 (WNA)** **[UF00147]**	217.22 ± 37.28
**1114** **[UF01114]**	133.74 ± 75.06
**1105** **[UF01105]**	156.97 ± 17.54
**1106** **[UF01106]**	168.45 ± 28.76
**1096** **[UF01096]**	150.04 ± 32.04
**1103** **[UF01103]**	276.88 ± 25.75
**1137** **[UF01137]**	81.92 ± 3.27
**1043** **[UF01043]**	96.26 ± 47.77

^a^ Strains in blue are lab strains and red are wild strains. Parenthesis indicate common names of lab strains.

^b^Data are culture doubling time in minutes calculated from the OD_600_ values plus or minus the SD of the replicate growth numbers from the beginning and end of exponential growth.

## Discussion

We found that wild strains of *B*. *anthracis* grew faster in laboratory media reaching higher CFU densities in 24 h than did laboratory strains during growth at 30°C in BHI, both serving to mimic optimal soil survival temperature and the nutrient rich nature of an animal carcass. As a group, sporulation levels and rates were also significantly higher in the wildlife outbreak strains versus the laboratory strains. While rich media such as BHI and growth at 30°C are not optimal for sporulation and growth of *B*. *anthracis*, they more closely resemble the conditions experienced at a LIZ soon after animal death. Nutrient poor media highly supplemented with ions optimized for sporulation could mask the subtle differences in nutrient acquisition and sporulation signals in a complex nutrient environment and are probably not as representative of an animal carcass as BHI. The reason sporulation media is used in laboratory production of spores is because it will force nearly the entire population of bacteria to sporulate. That being said, the observed faster growth and more efficient sporulation of wild strains was true across diverse sub-lineages of *B*. *anthracis* (Ames, Vollum, WNA, Sterne) and strains recovered from multiple wildlife and livestock species and outbreaks of varying intensity. These results support our group’s previous proteomics study, which suggested lab strains have lost the ability to sporulate as quickly as wild strains based on a higher abundance of proteins involved in sporulation than lab adapted strains [11]. Increased sporulation of wild strains is linked to the increased growth capacity and faster entry to stationary phase as discussed below. Interestingly, wild strains UF01096 and UF01103 (Vollum strains with similar behavior in our assays) were the identical MLVA-25 genotype, both recovered from dead deer during mixed outbreaks in West Texas but separated by 5 years [16]. For *B*. *anthracis* in a natural setting, rapid assimilation of nutrients and growth could be the difference between bacterial survival and death, as time between host bleed-out and desiccation might be quite short. Data from our growth analysis supported the findings in our direct CFU counting experiments. Laboratory adapted strains are known to quickly lose their ability to sporulate if grown on media that lacks a source of animal protein [27], and our data show that their growth rates in laboratory media are also slower than those of wild strains. Wild strains must be able to fine tune their metabolism and adapt quickly to nutrient availability or limitation by optimally regulating the genetic pathways involved in nutrient assimilation and sporulation [28]. Loss of ability to sporulate has been linked to modification of key genes involved in regulation of sporulation pathways [29]. The mechanisms underlying the slower growth rates we observed in laboratory strains have yet to be identified but we can speculate based on the previous proteomic work done comparing these same wild and lab strains [11]. The expression ratio of protein EA1 was ~20 times more prevalent in the wild strains versus lab. EA1 is an S-layer associated protein that is specifically expressed during stationary phase and takes the place of the logarithmic growth phase associated S-layer protein, Sap, on the outside of the cell before sporulation [30]. Diminished levels of certain ribosomal proteins (protein L4, L19, and L21) coinciding with increased levels of ribosomal protein L13 in wild versus lab strains indicate wild strain entry to stationary phase and the beginnings of sporulation as the lab strains continue to grow in logarithmic phase [11,31]. Of particular note is the ~14-fold higher levels of Fhs previously found in lab strains. Formate-tetrahydrafolate ligase creates 10-formyltetrahydrofolate, an important precursor of purines (e.g. adenine and guanine) and *N*-formylmethionyl-tRNA (i.e. usually required as the first amino acid during protein synthesis). Bacteria in stationary phase neither require as much purine nor new purine synthesis. Suffice it to say, wild strains make the transition to stationary faster because of the decreased lag, increased growth rates, and faster utilization of available nutrients thus inducing more rapid sporulation. If growth were not a component of the faster sporulation then lag, logarithmic and stationary phases would be the same when comparing the average growth curves. Decreased lag times, faster growth, and denser cultures all point to nutrient assimilation as the root cause of faster sporulation in wild *B*. *anthracis* strains.

Our results have implications for outbreak managers in the field. In enzootic areas of the US, including Texas and Montana, wildlife anthrax control relies on carcass identification and decontamination; burning is ideal and burial after surface decontamination with chlorine or formaldehyde is often used when burning is prohibited or dangerous (such as hot, dry summers in grasslands). This process can be challenging in remote landscapes and carcasses can sit for long periods of time before control measures can be implemented [8]. Our results suggest rapid efforts to search for and decontaminate LIZs (within 24 hours of death) are more likely to reduce spore concentrations at these LIZ sites.

Although sporulation does not directly contribute to virulence, it may be equally important to the infectivity of *B*. *anthracis* because it maintains infectious doses of organism in the environment. If sporulation was less efficient in wild strains, environmental persistence would not be sustained, and wildlife outbreaks would cease. Without sporulation, the cycle stops and without virulence the cycle stops. Demonstrating that growth and sporulation rates are higher in wild strains will enable more accurate modeling of environmental infectious spore doses.

## Supporting information

S1 Table Raw CFU, SFU, and spore percentages obtained from the sporulation experiments. Data is raw colony counts converted to CFU/SFU from dilution plating.
(XLSX)

S2 Table Raw OD_600_ measurements from the plate-based growth curve experiment. Data is the OD_600_ read every 15 minutes in duplicate.
(XLSX)

## References

[1] Carlson CJ, Getz WM, Kausrud KL, Cizauskas CA, Blackburn JK, Bustos Carrillo FA, et al Spores and soil from six sides: interdisciplinarity and the environmental biology of anthrax (Bacillus anthracis). Biological Reviews. 2018;93: 1813–1831. 29732670 10.1111/brv.12420

[2] Carlson CJ, Kracalik IT, Ross N, Alexander KA, Hugh-Jones ME, Fegan M, et al The global distribution of *Bacillus anthracis* and associated anthrax risk to humans, livestock and wildlife. Nat Microbiol. 2019; 1 10.1038/s41564-019-0435-4 31086311

[3] Halvorson HO. Two generations of spore research: from father to son. Microbiologia (Madrid, Spain). 1997;13: 131–48. 9253754

[4] Manchee RJ, Broster MG, Melling J, Henstridge RM, Stagg AJ. *Bacillus anthracis* on Gruinard Island. Nature. 1981;294: 254–255. 10.1038/294254a0 6795509

[5] Turner WC, Kausrud KL, Krishnappa YS, Cromsigt JPGM, Ganz HH, Mapaure I, et al Fatal attraction: vegetation responses to nutrient inputs attract foraging herbivores to infectious anthrax carcass sites. Proceedings of the Royal Society B: Biological Sciences. 2014;281: 20141785 10.1098/rspb.2014.1785 25274365 PMC4213624

[6] Hugh-Jones M, Blackburn J. The ecology of Bacillus anthracis. Molecular Aspects of Medicine. 2009;30: 356–367. 10.1016/j.mam.2009.08.003 19720074

[7] Alexander KA, Lewis BL, Marathe M, Eubank S, Blackburn JK. Modeling of Wildlife-Associated Zoonoses: Applications and Caveats. Vector-Borne and Zoonotic Diseases. 2012;12: 1005–1018. 10.1089/vbz.2012.0987 23199265 PMC3525896

[8] Blackburn JK, Mullins JC, Van Ert M, Hadfield T, O’Shea B, Hugh-Jones ME. The necrophagous fly anthrax transmission pathway: Empirical and genetic evidence from a wildlife epizootic in west Texas 2010. Vector-Borne and Zoonotic Diseases. 2014;14: 576–583. 25072988 10.1089/vbz.2013.1538

[9] Blackburn JK, McNyset KM, Curtis A, Hugh-Jones ME. Modeling the geographic distribution of *Bacillus anthracis*, the causative agent of anthrax disease, for the contiguous United States using predictive ecological [corrected] niche modeling. Am J Trop Med Hyg. 2007;77: 1103–10. 18165531

[10] Blackburn JK, Asher V, Stokke S, Hunter DL, Alexander KA. Dances with Anthrax: Wolves (Canis lupus) Kill Anthrax Bacteremic Plains Bison (Bison bison bison) in Southwestern Montana. Journal of Wildlife Diseases. 2014;50: 393–396. 10.7589/2013-08-204 24484485

[11] Leiser OP, Blackburn JK, Hadfield TL, Kreuzer HW, Wunschel DS, Bruckner-Lea CJ. Laboratory strains of *Bacillus anthracis* exhibit pervasive alteration in expression of proteins related to sporulation under laboratory conditions relative to genetically related wild strains. PLOS ONE. 2018;13: e0209120 10.1371/journal.pone.0209120 30557394 PMC6296524

[12] Lindeque PM, Turnbull PCB. Ecology and epidemiology of anthrax in the Etosha National Park, Namibia. Onderstepoort J Vet Res. 1994;61: 71–83. 7898901

[13] Wilson D. E., Chosewood L. C. Biosafety in microbiological and biomedical laboratories, 5th ed Atlanta, GA; 2007.

[14] ProMED-Mail. ANTHRAX, BOVINE—USA (03): (COLORADO). In: 20120809.1236375. 2012.

[15] Lista F, Faggioni G, Valjevac S, Ciammaruconi A, Vaissaire J, Le Doujet C, et al Genotyping of Bacillus anthracis strains based on automated capillary 25-loci multiple locus variable-number tandem repeats analysis. BMC microbiology. 2006;6: 33 10.1186/1471-2180-6-33 16600037 PMC1479350

[16] Mullins J. Combining genetic diversity and spatio-temporal data to characterize the spatial ecology of anthrax across multiple scales. University of Florida. 2013.

[17] Sterne M. Variation in *Bacillus anthracis*. Onderstepoort Journal of Veterinary Science and Animal Industry. 1937;8.

[18] Ravel J, Jiang L, Stanley ST, Wilson MR, Decker RS, Read TD, et al The complete genome sequence of *Bacillus anthracis* Ames “Ancestor”. J Bacteriol. 2009;191: 445–446. 10.1128/JB.01347-08 18952800 PMC2612425

[19] Smith NR, Gibson T, Gordon RE, Sneath PHA. Type cultures and proposed neotype cultures of somespecies in the genus *Bacillus*. Microbiology,. 1964;34: 269–272. 10.1099/00221287-34-2-269 14135533

[20] Van Ert MN, Easterday WR, Huynh LY, Okinaka RT, Hugh-Jones ME, Ravel J, et al Global genetic population structure of *Bacillus anthracis*. PLOS ONE. 2007;2: e461 10.1371/journal.pone.0000461 17520020 PMC1866244

[21] Blackburn JK, Goodin DG. Differentiation of Springtime Vegetation Indices Associated with Summer Anthrax Epizootics in West Texas, USA Deer. Journal of wildlife diseases. 2013;49: 699–703. 10.7589/2012-10-253 23778625

[22] Yang A, Mullins J, Van Ert MN, Bowen R, Hadfield TL, Blackburn JK. Predicting the geographic distribution of the Bacillus anthracis A1.a/Western North America Sub-lineage for the Continental United States: new outbreaks, new genotypes, and new climate data. American Journal of Tropical Medicine and Hygiene. 2019;In Press. 10.4269/ajtmh.19-0191 PMC7008322 31802730

[23] Minett F. Sporulation and viability of *B. anthracis* in relation to environmental temperature and humidity. Journal of Comparative Pathology and Therapeutics. 1950;60: 161–176. 10.1016/s0368-1742(50)80016-4 14814223

[24] Information NC for B, Pike USNL of M 8600 R, MD B, Usa 20894. Anthrax in animals. World Health Organization; 2008. https://www.ncbi.nlm.nih.gov/books/NBK310481/

[25] Dragon DC, Rennie RP. Evaluation of spore extraction and purification methods for selective recovery of viable *Bacillus anthracis* spores. Letters in Applied Microbiology. 2001;33: 100–105. 11472515 10.1046/j.1472-765x.2001.00966.x

[26] Davies DG. The influence of temperature and humidity on spore formation and germination in *Bacillus anthracis*. J Hyg (Lond). 1960;58: 177–186. 20475844 10.1017/s0022172400038250 PMC2134342

[27] Sastalla I, Leppla SH. Occurrence, recognition, and reversion of spontaneous, sporulation-deficient *Bacillus anthracis* mutants that arise during laboratory culture. Microbes Infect. 2012;14: 387–391. 10.1016/j.micinf.2011.11.009 22166343 PMC3306515

[28] Saile E, Koehler TM. Control of anthrax toxin gene expression by the transition state regulator *abrB*. J Bacteriol. 2002;184: 370–380. 10.1128/JB.184.2.370-380.2002 11751813 PMC139583

[29] Sastalla I, Rosovitz MJ, Leppla SH. Accidental selection and intentional restoration of sporulation-deficient *Bacillus anthracis* mutants. Appl Environ Microbiol. 2010;76: 6318–6321. 10.1128/AEM.00950-10 20639373 PMC2937495

[30] Mignot T, Mesnage S, Couture-Tosi E, Mock M, Fouet A. Developmental switch of S-layer protein synthesis in *Bacillus anthracis*. Molecular Microbiology. 2002;43: 1615–1627. 11952909 10.1046/j.1365-2958.2002.02852.x

[31] Ohashi Y, Inaoka T, Kasai K, Ito Y, Okamoto S, Satsu H, et al Expression profiling of translation-associated genes in sporulating *Bacillus subtilis* and consequence of sporulation by gene inactivation. Bioscience, Biotechnology, and Biochemistry. 2003;67: 2245–2253. 10.1271/bbb.67.2245 14586115

